# Protein 53 expression in a mixed Labrador subcutaneous lymphoma

**Published:** 2012

**Authors:** Annahita Rezaie, Abbas Tavassoli

**Affiliations:** 1 *Department of Pathobiology, Faculty of Veterinary Medicine, Shahid Chamran University of Ahvaz, Ahvaz, Iran; *; 2 *Department of Pathology, Faculty of Veterinary Medicine, University of Tehran, Tehran, Iran.*

**Keywords:** Subcutaneous Lymphoma, Dog, p53, T cell, Immunohistochemistry

## Abstract

An 11 year – old mixed female Labrador was presented with two masses in trunk and neck. The tumoral masses were excised and sent for histopathological and immunohistochemical analyses. Histopathological examination of masses revealed diffuse infiltration of small sized lymphoid cells in subcutaneous tissue which were intense around the blood vessels. More than 10% lymphoid cells were CD3 positive in the immunohistochemical staining and most of them were accumulated around vessels. Protein 53 (p53) expression was detected by brown nuclei in immunohistochemical staining. Subcutaneous lymphoma was diagnosed according to histopathological results. After 6 months the case was referred with multicentric lymphoma and based on the owner request euthanasia was performed. These findings emphasize on poor prognosis for tumors with p53 mutation.

## Introduction

Canine lymphoma is one of the most common malignancies and may affect the lymph nodes, bone marrow, liver, spleen, gastrointestinal tract, eye and skin. However, cutaneous lymphoma is relatively uncommon. Histopathologically, it can be divided into nonepitheliotropic and epitheliotropic forms.^1^^-^^3^ The nonepitheliotropic lymphoma is a heterogenous group of T and B cells lymphoma and is characterized by sheets and clusters of neoplastic lymphocytes. These are most often diffuse uncircumscribed infiltrates growing in the deep dermis or subcutis.^3^ Previously these were thought to be B cells, but now they have been shown to be predominately CD3 positive T cells. Epitheliotropic lymphoma is a subset of cutaneous T cell lymphoma and is the most common form of cutaneous lymphoma.^1^

The p53 is a tumor suppressor gene and wild – type p53 is present in all cell types, usually in low quantities, and has a short half - life that makes it difficult to be detected by immunohistochemical techniques. However, abnormal and nonfunctional p53 either caused by mutations of p53 gene may result in the synthesis of stable protein that is 10-20 fold longer half - life than that of wild - type p53, and so is detectable by immunohistochemistry. Absence of functional p53 may remove mechanisms that normally arrest the proliferation of transformed cells and disrupting the apoptotic response.^4^ Mutation of p53 was detected and reported in different canine carcinoma such as epithelial colorectal tumors,^4^ osseous tumors^5^ and cutaneous mast cell tumors.^6^

## Case History

An 11 year-old mixed female Labrador was presented to a private small animal clinic in Tehran, Iran. The primary clinical complaint was existence of two masses in trunk and neck. As the trunk mass was in mammary glands area so the first identification was mammary gland tumors. Abnormality noted on the initial physical examination was coughing. In radiographs, no other masses were seen. The tumoral masses were excised and after fixation in 10% neutral buffered formalin, then embedded in paraffin and stained by Hematoxylin and Eosin (H&E). 

Immunohistochemical staining was performed on paraffin embedded by using rabbit anti-human CD3 antibody (Dako, Copenhagen, Denmark), rabbit anti-human CD79 antibody and Polyclonal rabbit anti-p53 oncoprotein (Signet Laboratories, Dedham, USA) antibody (CM-1) as primary antibodies. Peroxidase conjugated anti-rabbit IgG were used as secondary antibodies.^7^

**Fig. 1:**
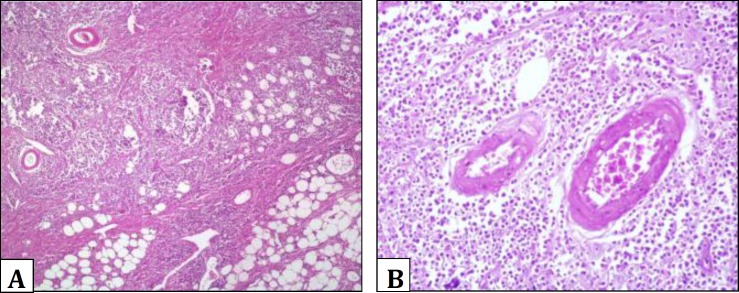
**A**. Histopathology section of trunk mass. Note monotonous population of neoplastic lymphoid cells which are infiltrated between adipocytes and around vessels (H & E, 4×). **B**. Part of Fig. 1A. infiltration of lymphoid cells is obvious around vessels (H & E, 20×).

Histopathologic findings demonstrated accumulation of a uniform mixture of large and small lymphocytes and single large pale-staining cells. They were among the lipocytes and around the vessels of subcutis (Fig. 1. A and B).

**Fig 2:**
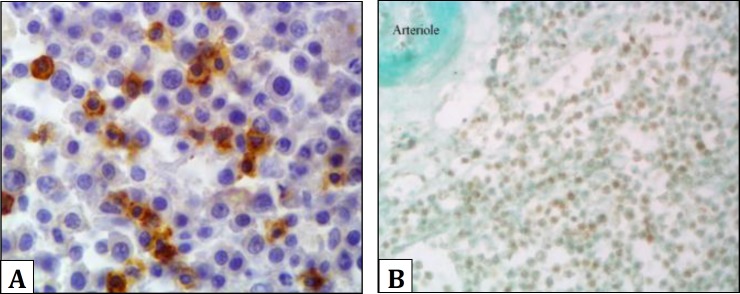
**A**. Immunostaining for CD3. Showing positive T cells lymphocytes (Immunohistochemistry staining, Hematoxylin counterstain, 100×). **B**. Immunostaining for p53. Positive p53 expression is clear by nuclear staining. (Immunohistochemistry staining, Fast green counterstain, 40×).

On CD3 staining, a high proportion of both the large and small lymphocytes stained positively, indicating T cell differentiation (Fig. 2A). On CD79 staining there was no stained cell which means that they are not B cell. In immunohistochemical staining for p53 detection, lymphocytes containing chromogen stain within their nucleus were considered to be positive (Fig. 2B). 

The immunophenotype of lymphoid and hematopoietic neoplasm can be precisely determined using a panel of monoclonal antibodies that recognizes B and T cell lymphocytes or other differentiation markers. Surface markers such as CD79a (B-cell lymphocyte marker) and CD3 (T-cell lymphocyte marker) are common and have efficacy in diagnosis of canine lymphoid neoplasm. The high reliability of these markers in routinely processed tissues was supported by different researches.^8^^-^^10^ The results of immunohistochemical staining of mast cell tumors using the CM-1 antibody in this study were similar to a previous study that evaluated p53 immunohistochemical staining prevalence of various canine tumors.^11^

Cutaneous lymphomas are subdivided into epitheliotropic and nonepitheliotropic varieties. The nonepitheliotropic subdivision which is called subcutaneous lymphoma in this report may be an expression of multicentric lymphoma or a variety of solitary or extranodal lymphoma. After six months the case was referred with respiratory difficulties. On clinical examinations there was enlargement of lymph nodes and two masses were seen in radiograph and multicentric lymphoma was diagnosed. According to the owner request euthanasia was performed and consent for necropsy was not given. Lymphoma tends to be a progressive disease, beginning and ultimately involving the lymph nodes. p53 expression in this case is a subsidiary for malignancies and using this marker may help for prognosis and recurrences.
